# Quarterly Abstract of Ophthalmological and Otological Literature

**Published:** 1881-11

**Authors:** E. J. Gardiner

**Affiliations:** Chicago


### Summary.

QUARTERLY ABSTRACT OF OPHTHALMOLOGICAL AND OTOLOGICAL LITERATURE. By Dr. E. J. GARDINER, Chicago.

ON THE CONTINUOUS ELECTRICAL CURRENT AS A THERAPEUTIC AGENT IN ATROPHY OF THE OPTIC NERVE, AND IN RETINITIS PIGMENTOSA. By R. Marcus Gunn, M.A., M.B.—(*Ophth. Hosp. Rep., Vol.* X., *P.* 2.)

*Method of application.*—Weiss' continuous current battery of 25 cells was used, fitted in the ordinary way with sponges, which were moistened in a solution of common salt. On employing it for the first time in any case, the anode was applied lightly over the closed lids of one eye, and the cathode on the opposite eye or temple. The power was gradually increased, beginning with five cells until a distinct flash was experienced by the patient, on making or breaking the circuit. The anode was kept over the eyelids, while the cathode was applied in turn to the top of the spine, to a point just behind the mastoid process, and to the supraorbital region of the same side, so as to determine which position gave the greatest light impression. This varied in different individuals, but in the majority of cases, the supraorbital region was selected.

This position (the supraorbital) being determined, the cathode was kept there for half a minute or so, then removed for a few seconds, and finally reapplied for the same period as before. It was next moved to the corresponding point on the opposite side of the head, and then to the closed eyelids of this side. In both these positions it was applied for a few moments. Both poles were now removed, and the cathode then applied where the anode had been, while the anode was applied to the supraorbital region, etc., as above. The same course was then gone through with the other eye, a slightly stronger current being finally used for a few seconds. The whole sitting lasted from five to eight minutes.

G. reports eighteen cases of atrophy of the optic nerve, treated in this manner. The following results were obtained: In case II, improvement doubtful; in cases I and X, temporary improvement; in cases V, VI, VII and XI there was marked improvement. All the other cases showed no improvement.

The continuous electrical current was tried in four cases of retinitis pigmentosa, with most satisfactory results. Case I when admitted V=in R. E. $=\frac{3}{200}$; L. E. $\frac{20}{200}$ with—36 V$=\frac{20}{70}$; when discharged May 10, R. E. V $<\frac{20}{70}$ with—40 V$=\frac{20}{70}$. L. E. V$=\frac{20}{40}$.

Case II.—Admitted Nov. 9, 1879. V=Jaeg. 4, barely with each. Dec. 20. Reads Jaeg. 1 fluently with R. E. barely with L. V$=\frac{20}{100}$ barely with each.

Case III.—Admitted Oct. 6, 1880. R. E. V $\frac{20}{100}$ with 2′0 D $\frac{20}{70}$ Jaeg. 1 at 12″. L. E. V$=\frac{20}{70}$ with—1′0 D. $\frac{20}{40}$ Jaeg. 1 at 12″. Discharged Dec. 20.

R. E. V$=\frac{20}{70}$: with—1′50 D$=\frac{20}{30}$ partly.

L. E. V$=\frac{20}{70}$: with—1′50 D$=\frac{20}{30}$ partly.

In Case IV the results were very satisfactory, but the amount of sight is not recorded.

### A Case of Simultaneous Subconjunctival Dislocation of both Crystaline Lenses, caused by the Kick of a Horse. By J. C. Wordsworth, F.R.C.S.—(*Oph. Hos. Rep. Vol.* X, *No.* II.)

A man, æt. 68, eight weeks before admission, had received a kick from a horse on his face, he was rendered unconscious for a few minutes. The bones of his nose were fractured, and both eyes were ruptured. "At the time of his admission, each crystaline lens was lying under the conjunctiva, at short distances from the cornea, a little above, and to the inner side of its margin—having escaped, through a laceration in the scleral coat of each eye, concentric to the upper and inner edge of the cornea." Good anterior chamber in both eyes; corneæ clear; pupils drawn upward and occupied by dark clots; eyes almost free from pain;

Six weeks after admission, the lenses were removed by division of conjunctiva over them. Very little irritation followed. Five months after injury was examined for glasses, when his vision was as follows:

R. E. with 10′50 D. V $\frac{20}{20}$ with 15 D. V=Jaeg. 1 at 7″.

L. E. with 10′50 D. V $\frac{20}{40}$ with 15 D. V=Jaeg. 1; slowly.

A CASE OF PRIMARY EPITHELIOMA OF THE LOWER EYELID. By George Lawson, F.R.C.S. (*Opth. Hos. Rep. Vol.* X, *P.* 2).

A man of 65 presented himself suffering from a primary epithelioma of the lower lid. He stated that it began as a small pimple, about three months previous it ulcerated and rapidly increased until it attained the present dimensions. Vertical diameter a little over three centimetres, horizontal diameter about three centimetres. It involved the greater part of the margin of the lower lid—it was a distinctly new growth—very solid when felt between the fingers—it could be raised from the subjacent tissues. The margin was hard and rounded, and the whole surface of the growth presented a coarse ulcerated surface, covered in parts with thick yellow purulent secretion. L. completely excised it, leaving an open wound to granulate and cicatrize. The patient has continued well since.

THREE CASES OF NEW FORMATION OF BLOOD-VESSELS IN THE VITREOUS. By W. Charney, M.A., M.D.; and L. Webster Fox, M.D. (*Opth. Hosp. Rep., Vol.* X, *P.* 2.)

Case I.—A young girl, æt. 14, a year ago noticed a black thread before her eye. On careful examination, the R. E. exhibited some small vessels which could be traced backwards to the lower margin of the discs, and which anteriorly formed a succession of loops and seemed to terminate near a glistening white mass in the vitreous.

Case II.—A girl æt. 19. R. E. Keratitis punctata—signs of previous iritis. In the L. E. was seen a floating vessel of a pale red color and peculiar spiral course, which could be traced forward to near the equator bulbi, then turning upward and outward, was lost in the vitreous; neither its point of origin or ending could be ascertained owing to the haziness of the media. While under treatment (mercurial) the caliber of these vessels gradually lessened, and eight months after the date of admission of the patient, they had entirely disappeared.

Case III.—Woman, æt. 51. R. E. downward from the disc was seen a grayish spot of connective tissue on the retina, from which a bunch of vessels extended upward and forward into the vitreous. Some presented free extremities, while others formed closed loops, returning to their place of origin, after having at their most anterior part formed a very fine and close spiral. They floated in the vitreous, moving with the motions of the eye.

VOLUNTARY NYSTAGMUS. By George Lawson, F.R.C.S. (*Opth. Hosp. Rep., Vol.* X, *P.* 2.)

L. reports the case of a gentleman who at will could set both his eyes into a rapid lateral motion. The movements were an exaggeration of what is seen in lateral nystagmus, they were so rapid however, that the margins of the cornea could not be defined.

*Des Indications de l'Iridectomie et de la Sclerotomie dans le Glaucome. Par le Docteur Ch. Abadie. Ann. d'Ocul. Mai, Juin.*

After reporting a few cases in which iridectomy was performed with no permanent relief to the patient, A. concludes that sclerotomy should be performed.

1st. In hæmorrhagic glaucoma.

2nd. In chronic simple glaucoma, characterized by the excavation of the disc, with no disturbance of the media, and by slight increase of the intra-ocular tension.

3rd. In cases of congenital hydrophthalmus with increase of tension and in which, owing to the deformity of the ciliary region, the excision of the iris may be followed by the rupture of the zonula, luxation of the lens, prolapse of vitreous, etc.

4th. In those rare forms of glaucoma which are characterized by a fatally progressive course, and which seem to continue in spite of the iridectomy. The advantage of sclerotomy in these cases, is that it can be often repeated until a pervious scar is obtained.

Iridectomy should be performed in those cases which exhibit a shallow anterior chamber, and where the iris and the lens are pushed forward.

Two Cases of Extraction of Splinters of Iron from the Vitreous. With Observations on the Diagnosis and Extraction of Steel and Iron Particles by Means of the Magnet. By Dr. H. Pagenstecher, of Wiesbaden. (*Arch. of Opth.*, *Vol.* X, *No.* 2.)

Case I.—A man, æt. 41, was struck upon the left eye by a piece of iron, ten hours before presenting himself. On the inner sclero-corneal margin there was a wound corresponding to which there was a wound in the iris. The iris was fastened to the wound; lens clear. On the 4th day, a yellowish nodule was seen projecting into the vitreous. As this indicated precisely enough the direction of the foreign body, the extraction was at once performed. An incision was made in the inner and lower part of the sclero-corneal margin, the corresponding portion of iris was cut out. A fine, bent probe was then introduced—between the lens and ciliary body—and the foreign body was touched; then a pair of simple tooth forceps were introduced in the same direction, and the foreign body having been grasped at the first attempt was removed. The border of the lens was wounded in the process of extraction. Vitreous did not escape. Healing took place without much reaction, though the opacity of the lens slowly increased. The cataract was removed five months later. With + 3, fingers were promptly counted at three meters.

In the 2nd case, Frölich's electro-magnet was used. The patient was a mason, 28 years old, who while breaking stone with an iron hammer, was struck by something on the left eye—Stat. Pres. Perforating wound of upper lid and corresponding to the same, a small linear one near the inner corneal margin in the sclera; cornea, iris and lens intact; blood on the floor of the anterior chamber. In the middle of the vitreous was seen a pretty large air-bubble, and downward and outward there was a striped opacity in the vitreous which terminated in a considerable hæmorrhage on the floor of the vitreous ; pain during the night. Next day cornea and aqueous hazy ; iris swollen, deeply injected and painful. On the 4th day panophthalmitis seemed likely to ensue. With patient fully under the influence of chloroform, P. made a small incision in the sclera, in the situation of the previously observed blood cot. Through this the sound of the electro-magnet was introduced and moved around. A loud click of the foreign body against the sound was heard. Two ineffectual attempts were made to extract the foreign body ; finally, having enlarged the sound, a rough angular splinter of iron two millimetres wide and one millimetre thick was drawn out. A slight loss of vitreous tinged with blood followed ; wound was closed with antiseptic bandage. Everything continued well for a few days, but soon a lingering irido-choroditis set in, which led to the shrinking of the globe. For locating the foreign body P. recommends the use of a magnetic needle such as described by Pooley, only Pagenstecher allows the needle to rotate as in a compass on a fine needle. Attention is drawn to the fact that if the media are clear and the foreign body is movable, in rendering the eye magnetic by touching it with the electro-magnet, the foreign body will be seen to follow the movements of the magnet. If this experiment fails, it is likely that the foreign body is imbedded in the tunics of the eye, and then the examination with the magnetic needle is to be resorted to. The experiments performed upon rabbits, with Frölick's sound were generally successful ; if the reaction produced by the foreign body was not too great. With regard to the operation in man the prognosis is not so favorable. P. says, however, that "the introduction of the magnet for the purpose of removing fragments of steel and iron from the eye, and for their diagnosis, is a considerable step in advance, and we are now in a condition to save many an eye which would otherwise irreparably be lost."

CLINICAL CONTRIBUTIONS. By Dr. E. L. Holmes.—(*Arch. of Oph.*, *Vol.* X, *No.* II.)

*A Case of Aneurismal Tumor of the Orbit.* The patient, a lady of 40, was suddenly seized with a most violent pain in left rbit. An enormous oxopthalmus at once followed.

When H. saw the patient auscultation and palpation revealed the sound and impulse peculiar to aneurism—redness and œdema of exposed ocular conjunctiva; cornea had lost its sensitiveness, it was only kept moist by artificial means, and showed signs of speedy and complete necrosis; condition of lens, vitreous and fundus could not be determined. It was deemed best to ligate the carotid artery. After two days, however, the aneurismal symptoms diminished. Five weeks later the sounds had spontaneously ceased, with great decrease of swelling and pain. At the end of sixty days after attack, the lids rested partially closed over an atrophied globe. Two years later the patient died suddenly at the breakfast table, without the slightest premonition.

*Case of Persistent Hyaloid Canal.*—A boy, 17, presented himself suffering from a high degree of amolyopia in the R. E. Examination revealed "a remarkable white object, resembling a wine bottle, with its body, neck and head reaching forward almost to the center of the post. cap. of the lens."

*A Case of Traumatic Aneridia.*—A man, æt. 53, had received (two months previous to examination) a severe blow on the right side of face and eye. No traces of iris could be found. With a proper direction of the light from the mirror, a clear, dark ring could be seen enclosing a black ring (the periphery of the lens) within which was the ordinary red reflex. The ciliary processes were not visible as regular elevations, but simply as a dark ridge just behind the border of the cornea; changes of form of the lens and of the ciliary processes could not be discerned during efforts of accommodation. There was no trembling of the lens.

ON QUININE AMAUROSIS. By Dr. H. Knapp.—(*Arch. of Opth., Vol. X, No. 2.*)

Blindness was produced in the three cases by quinine poisoning. The restoration of central acuteness of vision was complete in first case. It was incomplete in the second and third. K. considers the prognosis favorable even in advanced cases, there being thus far no case of permanent blindness on record.

Du Traitement de la Keratite Instertitielle, et de la Sclero-keratite.—Par l'Iridectomie ; par le Dr. Galezowski. (*Rec. d' Opth.*, *series* 3, *No.* 7.)

G. thinks that in those cases of intersticial keratitis where the iris becomes implicated, iridectomy should be performed. He reports several cases in which this operation stemmed the disease when other treatment had failed to do so. His reason for operating is that, "when the iris becomes adherent to the capsule after a time it disturbs the circulation in the anterior lymphatic canals, thus depriving the cornea of its nutrition."

An Operation for Prominence of the Auricles. By Dr. Edward T. Ely.—(*Arch. of Otol.*, *Vol.* X, *No.* 2.)

The description of the operation being a short one I will quote Dr. E. "An incision was made through the skin along the entire length of the furrow formed by the junction of the auricle with the side of the head posteriorly. This was joined at each end by a curved incision carried over the posterior surface of the auricle, and the skin and subcutaneous tissue included by these incisions were dissected off. Two incisions, nearly parallel to the prominence were then carried directly through the cartilage and an elliptical piece of the latter measuring $1\frac{1}{8}$ inch by $\frac{1}{3}$ inch, was removed. The piece of excised skin was considerably larger than this. The edges of the wound were then united by ten sutures, of which seven included only the skin, while three passed through both skin and cartilage. Owing to the natural folds of the cartilage, it was impossible to secure perfect coaptation of the anterior surface of the auricle, and a small piece was here left to heal by granulation. The dressing consisted of absorbent cotton and a bandage. . . . Healing ensued without accident.

Bilateral Rudimentary Auricle with Absence of the External Auditory Canal. By Dr. H. Knapp.—*Arch. of Otol.*, *Vol.* X, *P.* 2.)

A man, age 30, of healthy parentage, was born with a rudimentary auricle on both sides. "The auricle presents a curved, crook-like ridge of skin, which in the upper curved half contains cartilage, whereas the lower half, which has the feel of the lobule, consists only of skin. Behind the rudimentary auricle there is a flat, round depression of half an inch in diameter, bordered posteriorly by a well developed mastoid process. Placing the finger in the depression, the movements of the lower jaw are felt as distinctly and in the same manner as when the finger is placed in a healthy auditory canal. The depression behind the ear therefore, corresponds to the external meatus." The whole head, in particular the pharynx, nose and jaws are well developed. A catheter can be introduced into the tubes and the stream of air will be heard to penetrate into the tympanic cavities, when a stethoscope is applied both in front and behind the rudiment of the auricle. . . . He understands loud voice with some difficulty at the distance of five or six feet. A physician of Philadelphia, thirteen years ago, unsuccessfully attempted to open the passage.

---

GOWERS ON PARALYTIC CHOREA.—Five cases in children of ages varying from seven to fourteen years, three of whom were girls, formed the basis of a paper read by Dr. Gowers at last year's meeting of the British Medical Association. They illustrate that form of chorea in which the muscular weakness is the prominent symptom, the twitching and inco-ordination of voluntary movements being slight. In regard to such cases, Dr. Gowers remarks, rarely slight clonic spasm occurs in the affected arm at first, and subsequently passes off. The loss of power may be very great, as it may be much less than the loss of use of the limb would suggest. In these cases there is inaction rather than paralysis ; usually one arm only is affected, and there is no paralysis of face, tongue or leg. Both arms may be affected, but one is always the weaker. On careful watching, clonic twitching is to be observed, though often very slight. The course of such cases is very tedious, but they have not been seen to pass into severe general chorea. The reported cases all recovered under the use of liq. strychniæ.—*London Medical Record.*

